# Spinal Cord Compression as the First Presentation of Primary Hyperoxaluria in a Patient With Kidney Failure: A Case Report and Literature Review

**DOI:** 10.1016/j.xkme.2024.100932

**Published:** 2024-11-14

**Authors:** Marwa Kliea, Mohammad Alsultan, Safaa Qatleesh, Yousef Haroun, Osama Abdul Aziz, Kassem Basha

**Affiliations:** 1 Department of Neurology, Damascus University-Faculty of Medicine, Damascus, Syria, MA; 2 Department of Histopathology, Damascus University-Faculty of Medicine, Damascus, Syria

**Keywords:** Calcium oxalate, kidney failure, mass-like lesions, primary hyperoxaluria, spinal cord compression

## Abstract

A 50-year-old woman with kidney failure complained of back pain and an inability to walk. The medical history included hypothyroidism, nephrolithiasis, and resistant anemia aligned with several transfusions. The examination showed hepatosplenomegaly, lower limb weakness, absence of reflexes, and lack of sensations with a sensory level T6. Laboratory results showed hypercalcemia with suppression of parathyroid hormone levels. Magnetic resonance imaging showed vertebral fractures and mass-like lesions that compressed the spine at T4, T9, L4, and L5. Vertebral and bone marrow biopsies showed calcium oxalate (CaOx) depositions. Here, we reported a rare case of primary hyperoxaluria (PH) in a patient with kidney failure who presented with spinal cord compression caused by vertebral fractures and mass-like lesions. We summarized a literature review of PH patients with spinal cord compression, which showed only 3 cases. The multiorgan CaOx infiltration in this patient also caused resistant anemia, hepatosplenomegaly, extensive bone lesions, hypoparathyroidism, hypothyroidism, and hypercalcemia. The overdiagnosis of renal osteodystrophy and the negative family history could delay the diagnosis of PH in patients with kidney failure. Thus, clinicians should always consider PH in the differential diagnosis of kidney failure patients with stone events given that the early diagnosis of PH could be lifesaving.

## Introduction

Primary hyperoxaluria (PH) is a metabolic disease with an estimated prevalence of 1 to 3 per million people and an incidence rate of about 1 per 100,000 births.^1^^,^^2^ It is a rare autosomal recessive disorder characterized by errors in metabolism leading to excessive production of calcium oxalate (CaOx).^3^^,^^4^ Oxalate is a metabolic end product that forms a poorly soluble salt of CaOx, which is mainly excreted by kidneys. Increased excretion and deposition of CaOx in renal tubules and parenchyma leads to tubular toxicity, urolithiasis, nephrocalcinosis, and eventually progression to kidney failure.^3^^,^^4^

There are 3 types of PH. Type 1 is caused by variants of AGXT that encode the hepatic peroxisomal enzyme alanine glyoxylate aminotransferase. PH type 1 is the most common, accounts for approximately 70%-80% of cases and is the most severe form with more rapid progression to kidney failure.^5^^,^^6^

PH type 2 is caused by variants in the gene that encodes the cytosolic enzyme glyoxylate reductase/hydroxypyruvate reductase (GRHPR). It accounts for approximately 10% percent of cases.^5^^,^^6^ PH type 3 is caused by variants of HOGA1 that encode the liver-specific mitochondrial 4-hydroxy-2-oxoglutarate aldolase enzyme. It is the mildest form of PH and appears to account for approximately 5%-10%.^5^^,^^6^

Secondary hyperoxaluria develops by increased intestinal absorption (short bowel syndrome or inflammatory bowel disease) or increased dietary intake of oxalate. The lack of bacteria that ferment oxalate like *Oxalobacter formigenes* secondary to antibiotic use has been reported as a cause.^7^ In the differential diagnosis of secondary form, determination of intestinal oxalate absorption and stone analysis may be helpful.^7^ Pure CaOx monohydrate (whewellite) stones are usually seen in PH, whereas mixed (whewellite and weddellite) stones are seen in the secondary form.^7^

Here, we report a spinal cord injury as the first presentation of PH in a woman with kidney failure. The diagnosis by 2 different biopsies from the vertebrae and bone marrow showed CaOx depositions, which corresponded with systemic oxalosis.

## Case Report

A 50-year-old woman was admitted to the neurology department with back pain and an inability to walk. She had intermittent back pain for 18 months, which spread to her lower limbs alternately. Over the past 5 months, the back pain gradually increased to be severe and constant. Two weeks before admission, the patient became unable to walk and lacked sensation up to the xiphoid process with numbness but no history of sphincter incontinence.

The medical history included a gallbladder excision (at the age of 40 years old) and 2 episodes of kidney nephrolithiasis, one of them with lithotripsy when she was 42 years old. At age 46 years old, the patient was diagnosed with hypothyroidism, hypertension, and kidney failure with kidney replacement therapy (programmed hemodialysis [HD], 2 sessions per week). The patient was treated conservatively for kidney failure and did not receive a kidney biopsy or a previous evaluation for kidney transplantation. Also, the patient described longstanding anemia, lasting from the HD initiation, that was resistant to iron supplements and erythropoietin replacement aligned with several blood transfusions. She received levothyroxine, methyldopa, folic acid, erythropoietin, and iron supplements.

General examination showed pallor of conjunctiva, spleen and liver hypertrophy, and pretibial edema grade 2. In a neurological examination, the patient was conscious and oriented, and no cranial nerve injury was observed. Upper limbs showed normal strength, tone, and reflexes. Lower limbs showed decreased strength (3+/5), normal to flaccid tone, and absence of reflexes with bilateral flexion of planter reflexes. A decreased sensation with sensory level T6 was observed.

**Figure 1:**
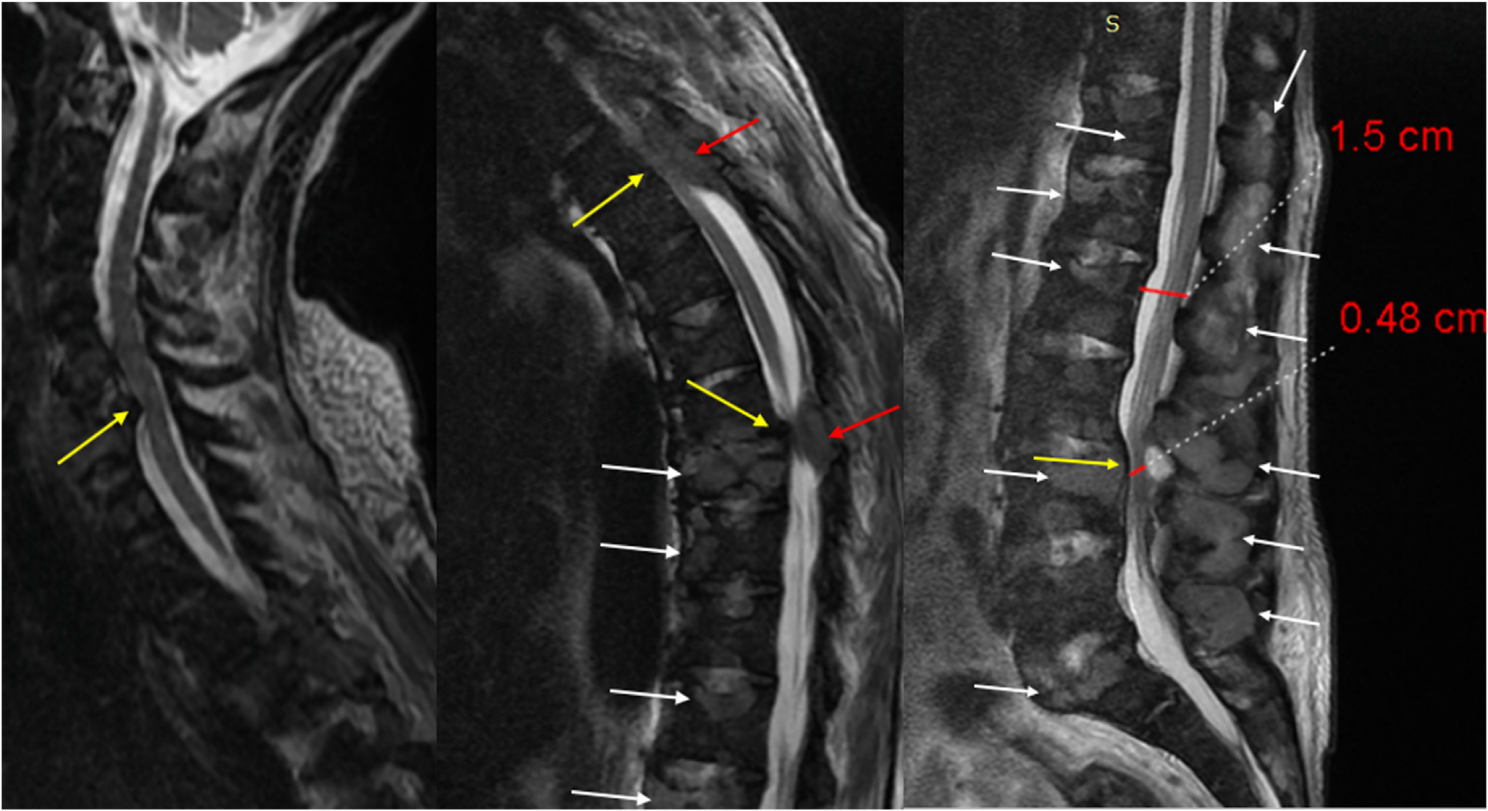
MRI (cervical, thoracic, and lumbar) without contrast; T2 sequence shows the following:1)Vertebral and spinous processes deformities and compressions (white arrows).2)Spinal cord compression and spinal canal stenosis on cervical, thoracic, and lumber spine (yellow arrows)3)Epidural mass effect on T4, T9 (red arrows).

The laboratory tests showed anemia, hypercalcemia with suppression of parathyroid hormone (PTH) levels, hypoproteinemia, and kidney failure (Table 1). Magnetic resonance imaging (MRI) of the thoracic and lumbar spine without contrast (Fig 1) revealed multiple lesions that were isointense in the T1 sequence and iso- to hyperintense in the T2 sequence distributed in several vertebrae. These deformities represent vertebral lytic and erosion, which compresses the spine on T4 and T9, and another compression on L4-L5. Two lesions that were iso- to hyperintense in the T2 sequence (epidural infiltrations or mass-like lesions) also compress the spine on T4 and T9-T10. MRI of the cervical spine (Fig 1) with the same vertebral characters showed vertebral compression of the spine on C6-C7.

**Table 1:** Laboratory Investigations

Laboratory Tests at Admission								
WBC	L/N	RBCs	Hb/Ht	Mcv	PLT	Ur/ Cr	TP/ ALB	ALT/ AST
5.8	22/67	3.34	9.4/28.7	85	169	112/8.3	3.8/2.7	10/41
TB/DB	ALP	Ca	Corrected Ca	P	Na/ K	UA	INR	CRP /ESR^a^
0.13/ 0.1	109	12.07	13.1	5.7	136/ 5.13	4.1	1.1	8/ 35
Iron	Ferritin	LDH	CK	PH	Pco_2_/Hco_3_	TST	PTH	TSH
33	569.6	235	62	7.28	30/ 14	Neg	2.81	4.6
**Pleural Effusion Analysis**								
WBCs	RBCs	Glu	TP	ALB	Cholesterol	LDH	ADA^b^	
30/mm^3^	350/mm^3^	97	1.2	0.5	33	62	2.1 (neg)	

Abbreviations: ADA, adenosine deaminase; ALB, albumin; CRP, C-reactive protein; ESR, erythrocyte sedimentation rate; Glu, glucose; Hb/Ht, hemoglobin and hematocrit; LDH, lactate dehydrogenase; L/N, lymphocytes and neutrophils; MCV, mean corpuscular volume; PLT, platelets; RBC, red blood cells; TST, tuberculin skin test; UA, uric acid.

a CRP (up to 5 mg/L), ESR (up to 30).

b ADA (18.46–27.50 U/L).

The surgery was performed under general anesthesia. A dorsal incision and laminectomy were performed at the level of thoracic vertebrae (T4, T5, T6). Vertebrae were friable and eroded with an infiltrating lesion over the meninges, where the spinal cord was decompressed. Another midlumbar incision at the level of the lumbar vertebrae (L4, L5) was performed, and the vertebrae were also found to be eroded. The spinal cord and nerve roots on both sides were decompressed. The surgeon could not do spinal fixation and biopsies were taken from the vertebrae and laminae and sent for pathology.

When the pathologic results need at least 20 days in our university hospital, further investigations have been evaluated to rule out other disorders. Based on all these findings (MRI characters, hypercalcemia with PTH suppression, and hepatosplenomegaly), there were broad differential diagnoses such as multiple myeloma, metastasis, infections (such as tuberculosis), amyloidosis, and metabolic infiltration disorders.

**Figure 2:**
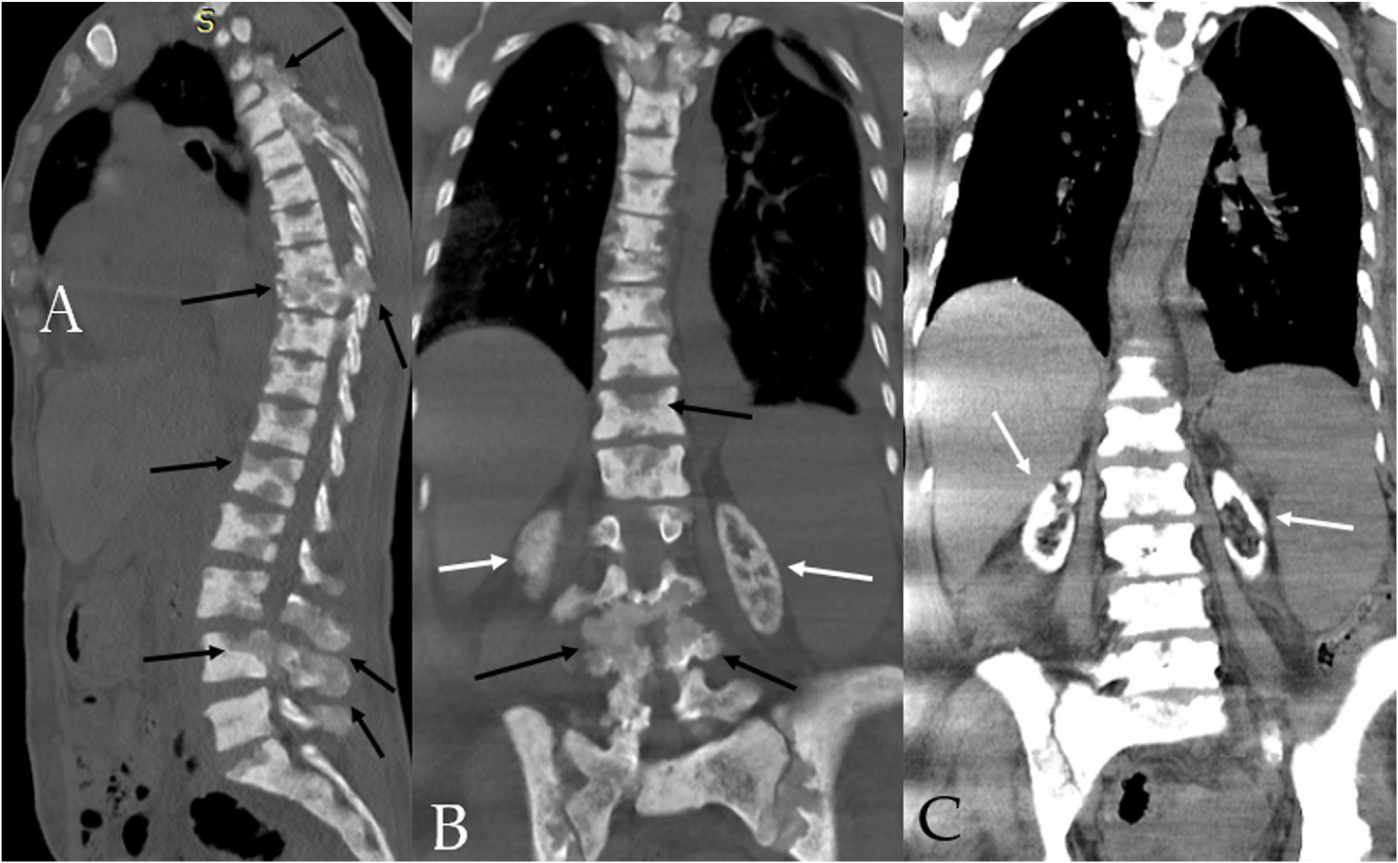
CT scan; bone (A+B) and abdominal (C) windows shows the following:1)Diffused lytic lesions along all vertebrae with vertebral erosions and destruction of vertebral bodies and spinous processes (black arrows).2)Cortical kidney calcifications (white arrows)3)Hepatosplenomegaly.

To search for a malignant source, a computed tomography (CT) scan was obtained (Fig 2), which showed pleural effusion, hepatosplenomegaly, calcifications, and lytic lesions in vertebrae, calcifications in soft tissues adjacent to the vertebrae, and calcifications in both kidneys (cortical calcifications).

On neck, abdomen, and pelvic ultrasounds, the thyroid gland showed the following: the left lobe showed well-defined, parallel echogenic, cystic, and calcified nodule (0.6 × 0.7 cm), and the right lobe was normal. The liver showed homogenous and regular borders without focal lesions and measured 170 mm on the axis parallel to the right kidney (hepatomegaly). The gallbladder was absent with no enlargement in the biliary duct. The spleen was homogenous and measured 180 mm (splenomegaly). Kidneys were atrophic without differentiation aligned with many stones’ shadows and cortical calcifications. The genital tract was normal. Echocardiography showed normal atriums, ventricles, valves, and pericardium, with no signs of cardiac amyloidosis.

Mammography could not be performed because the patient was unable to stand but breasts were normal on ultrasound. Upper and lower gastrointestinal endoscopies were normal. Pleural aspiration showed transudate fluid (Table 1) that was negative for TB (by a gene expert), abnormal cells, and cultures. Furthermore, tracheobronchoscopy was normal, and bronchoalveolar lavage was negative for gene expert TB, abnormal cells, and cultures.

Serum protein electrophoresis showed hypoproteinemia, hypoalbuminemia, hypogammaglobulinemia, and no monoclonal gammopathy. Regarding the anemia study, the blood smear did not show abnormal cells. Sternal and bone marrow biopsies showed hypocellularity and no abnormal cells.

**Figure 3:**
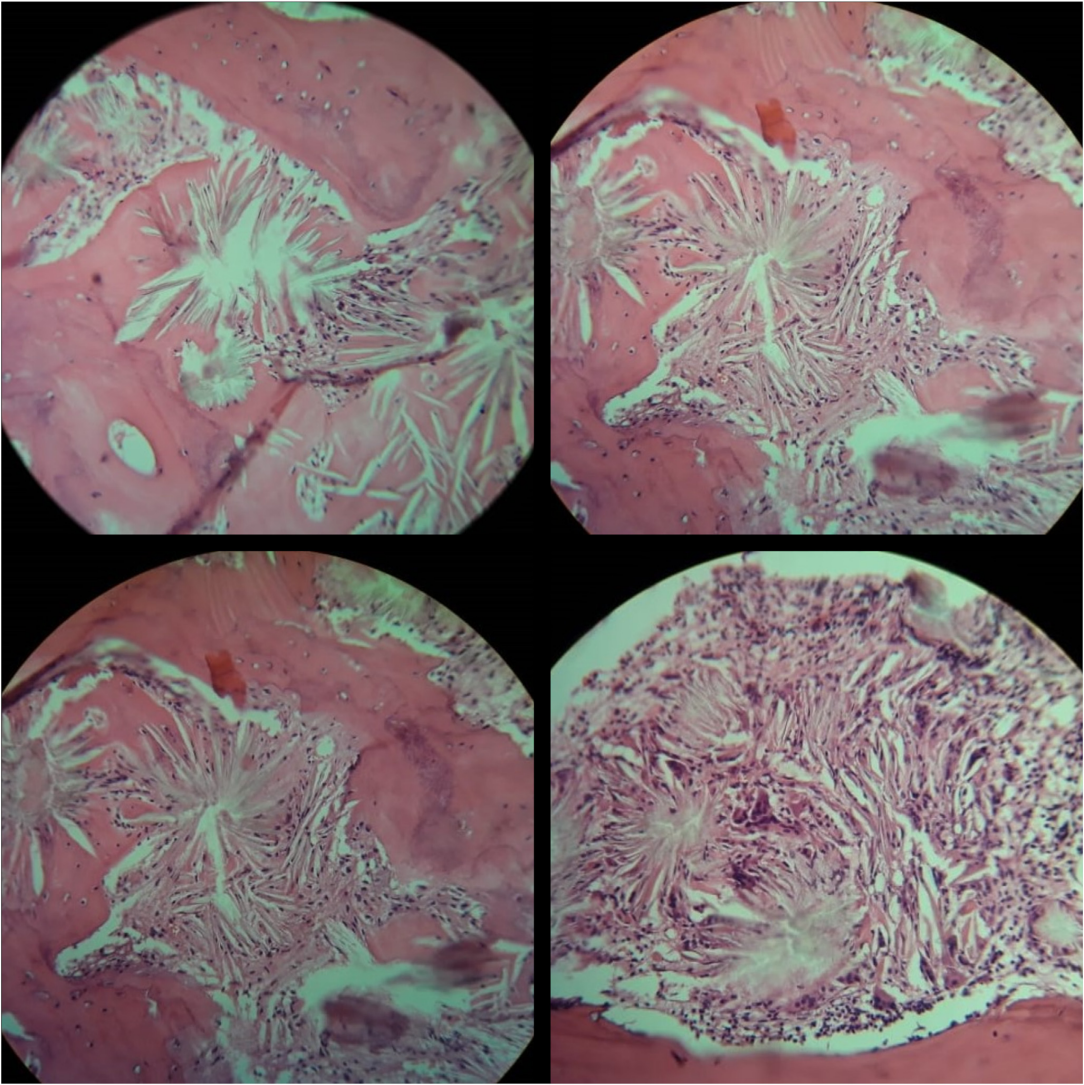
Vertebral and bone marrow biopsies (H&E stain, ×200). A significant number of bright needle-shaped oxalate crystals are noted within osseous tissue.

**Figure 4:**
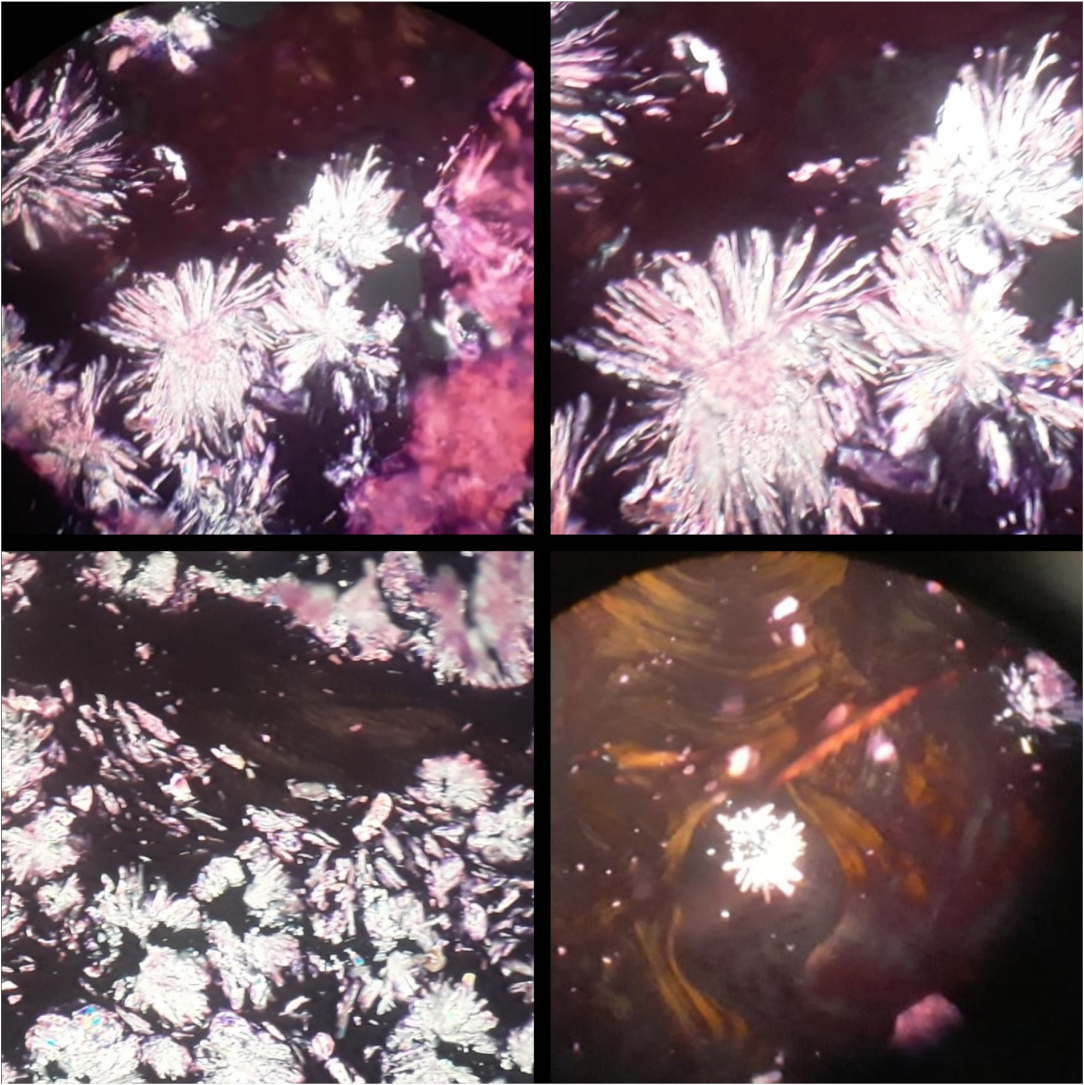
Vertebral and bone marrow biopsies (polarized light). Oxalate crystals showed positive birefringence.

Finally, pathologic study results from vertebral and bone marrow biopsies showed necrotic osseous materials involved by depositions of CaOx crystals, which form a radial pattern that encircled and engulfed by epithelioid and giant cells foreign body reaction (Fig 3). Fan-shaped crystals with radial bands are birefringent under polarized light (Fig 4). The same depositions of CaOx showed in bone marrow biopsies with reduced foci of trilineage hematopoietic elements. Immunohistochemistry stains were applied for further evaluation (negative for EMA, CK7, CK5/6, ER, TTF1, and MPO; positive for vimentin; less than 3% Ki67 positive), that ruled out the evidence of metastases from breast and lung cancers. The final diagnosis was systemic CaOx depositions with foreign body reactions.

The diagnosis of oxalosis was made by bone biopsies. Furthermore, the genetic test and measurement of plasma oxalate (Pox) should be applied. However, when the hospital did not cover the costs of these tests, the patient and their relatives refused applying tests because of high costs.

Repeated thoracic and lumbar MRIs showed a vertebral collapse in T4 and T5. The patient was discharged on conservative management with back fixation and could walk with help. Unfortunately, 2 months later the patient’s status deteriorated, and she died.

## Discussion

PH could affect any organ by CaOx infiltration; however, neurologic involvement was scantily reported in the literature. In Table 2, we summarize oxalosis patients with neurologic involvement, which is defined by either neurologic symptoms or biopsies showing CaOx without neurologic symptoms, and thirteen patients manifested neurologic involvement, with both the peripheral and central nervous systems were involved.^3^^,^^8^, ^9^, ^10^, ^11^, ^12^, ^13^, ^14^, ^15^, ^16^, ^17^, ^18^

**Table 2:** Literature Review for Oxalosis Patients With Neurologic Involvement, Either Neurologic Symptoms or Biopsies

Author (y)	Age/Sex	Presentation/Neurological finding	Investigations/Imagining	Diagnosis (Pathology/Genetics)	Treatment	Outcome
M. T. Haqqani et al^8^ (1977)	50 y/F	- Anorexia, vomiting, and anuria - No neurologic symptoms	NA	- Autopsy: crystalline deposition in vessels, heart, meninges, and brain	PD	Died
D. T. D. Hughes et al^9^ (1959)	5 w/M	Twitching of the left hand, jerky rolling of the eyes, and generalized convulsions.	NA	- Autopsy: crystals found in kidneys and brain	NA	Died
Ardemani G et al^11^ (2017)	2 m/M	Left arm convulsions and fixed eye deviation.	- Cranial ultrasound: high signal intensity of thalami and putamina - Ophthalmoscopy: retinal crystals in both maculae	PH1	NA	NA
Bilbao J et al^13^ (1976)	61 y/F	- Numbness of hands and feet, progressive muscle weakness. - Muscles atrophy of both hands and distal weakness in all extremities. - Distal sensory loss extending up to the lower forearm and to above the ankles. - All tendon reflexes were absent and no response to plantar stimulation.	NA	- Autopsy: massive crystal depositions were found in the kidney, peripheral nerves, bone, aorta, testes, and heart - Few crystals depositions in the posterior pituitary gland, pancreas, and cartilage of a pulmonary hamartoma	HD	Died
Ahmed A et al^14^ (2021)	4 m/M	- Seizures - Anemia, metabolic acidosis, hyponatremia, hyperkalemia, ESKD	- Renal ultrasound: diffuse echogenicity and bilateral calcifications. - Crystalline retinopathy	- Autopsy: extensive crystal depositions in kidneys Genetic study: PH 1 (AGXT)	HD, pyridoxine, 2 liver transplants	Died
Farrell J et al^15^ (1997)	22 y/F	- Progressive weakness and shortness of breath with occasional vomiting - ESKD, bilateral livedo reticularis - Decreased central vision - Numbness and bilateral sensory loss below the ankles	- Ophthalmoscopy: extensive depositions of crestas in and around the macula - Abd x-ray: diffuse nephrocalcinosis	- Skin biopsy (livedo reticularis lesions): a crystalline material infiltrating capillary walls and interstitium - Genetic study: PH 1 (AGXT)	HD, SLKT	Improved
Moorhead PJ et al^16^ (1975)	23 y/M	- Painful paresthesia and weakness in legs - Diminished tendon reflexes in all extremities	NA	- Autopsy: heavy deposits of CaOx in renal, retia, testes, myocardium, aorta, and arterioles in all organs except the brain and meninges - Spare deposits in bronchial cartilage, bone marrow, trabeculae, parathyroids, pancreas, prostate, gastric submucosa, and choroid plexus - Both sciatic and peroneal nerves showed CaOx deposits	HD	Died
Berini S et al^17^ (2014)	24 y/M	- Rapidly progressive numbness and weakness affecting the upper and lower limbs - Sensory ataxia, pain, and severe muscle cramping - Areflexic with a stocking-glove distribution of sensory loss to all modalities	- NCS: severe, diffuse mixed axonal and demyelinating polyradiculoneuropathy	- Sural nerve biopsy: inclusions typical for CaOx - Genetic study: PH 1 (AGXT)	Pyridoxine, CVVH, HD, SLKT	Improved
Bouraoui H et al^12^ (2008)	20 y/M	- Ophthalmological damage - Peripheral sensory polyneuropathy - Pericardial calcifications and abdominal pain	- Abd x-ray: calcified left kidney, high density of bone - CT: massive nephrocalcinosis, calcification of the digestive walls and arterial walls	- Known PH1 since age 12 y	NA	NA
	10 y/F	- Left ventricular failure - Severe sensorimotor peripheral polyneuropathy - Diarrhea	- CT: calcifications of the digestive wall.	- Known PH	NA	NA
Dieudonné H et al^3^ (2018)	57 y/F	- ESKD - Progressive weakness, gait disorders with repeated falls, and dysuria - Myelopathy syndrome; impaired proprioceptive and vibratory sensations on T3 level - Neck flexion triggered limb paresthesia (Lhermitte’s sign)	- MRI: cervical, thoracic (C7-T4), and lumbar (L2-L3) mass-like lesion with spinal cord compression	- Bone biopsy: massive crystal deposition. Genetic study; PH 1 (AGXT).	Pyridoxine, HD, on the waiting list for SLKT	Improved
Munarriz L et al^10^ (2019)	17 y/M	- ESKD - Pain in the spine and large joints of the ankles, knees, and shoulders - Progressive deformity of the hands, spontaneous fractures - Chronic liver disease and portal hypertension	- MRI: flattening of D8-D9. - Abd x-ray: nephrocalcinosis and renal lithiasis	- Bone biopsy: the presence of CaOx - Genetic study: PH 2 (GRHPR)	PD and HD	NA
Perrin P et al^18^ (2022)	67 y/ F	- ESKD, fracture - Spinal cord compression (back pain, impaired mobility) - Aortic stenosis	- CT: reactive bone resorption areas adjacent to CaOx	-Bone, soft tissues, and node biopsies: metabolic lesion of CaOx crystals. - Genetic study: PH 1 (AGXT)	Dialysis, SLKT	NA

Abbreviations: Abd, abdominal; CT, computed tomography; CaOx, calcium oxalate; CVVH, continuous veno-venous hemofiltration; ESKD, end-stage kidney disease; F, female; HD, hemodialysis; m, months; M, male; MRI, magnetic resonance imaging; NA, not available; PD, peritoneal dialysis; PH, primary hyperoxaluria; SLKT, simultaneous liver–kidney transplantation; NCS, nerve conduction studies and electromyography; US, ultrasound; w, weeks; y, year.

Only 1 case by Haqqani et al^8^ reported multiorgan CaOx infiltration including brain without neurologic symptoms in an autopsy of a 50-year-old woman. The remainder of the mentioned cases presented with neurologic symptoms. Three patients presented with seizures.^9^^,^^11^^,^^14^ Six patients presented with peripheral neuropathy, which manifested as numbness, weakness, sensory loss, sensory ataxia, and absence of tendon reflexes.^12^^,^^13^^,^^15^, ^16^, ^17^ CaOx crystal depositions were found in peripheral nerve biopsies in 3 patients.^13^^,^^16^^,^^17^

On the other hand, only 3 patients manifested spinal cord symptoms due to compression.^3^^,^^10^^,^^18^ Loza Munarriz et al^10^ and P Perrin et al^18^ reported 2 patients with myelopathy syndrome due to vertebral fractures that compressed the spinal cord. Bone biopsy showed CaOx crystal deposition in both patients.^10^^,^^18^ Genetic studies showed PH2 (GRHPR) in the first and PH1 (AGXT) in the second patient.^10^^,^^18^ Dieudonné Y et al^3^ reported a 57-year-old woman with spinal cord compression because of mass-like lesions in the cervical, thoracic, and lumbar areas. In addition, bone biopsy showed massive crystal deposition, and the genetic study classified the condition as PH1 (AGXT).^3^

Our patient had 2 etiologies: vertebral destruction and mass-like lesions. MRI showed epidural mass-like lesions that compressed the spine on T4 and T9-T10. Also, multiple vertebral osteolytic lesions and vertebral compression in T4, T9, L4- L5, and C6-C7 were noted on MRI. Pathologic studies showed necrotic osseous materials involved in depositions of CaOx crystals in vertebral and bone marrow biopsies. However, a genetic study could not be performed.

In all previous reports of hypercalcemia induced by PH, the bone biopsy was associated with macrophage infiltration that surrounded CaOx crystal deposits. Also, hypoparathyroidism was reported in all these cases, except 1 case reported by Brancaccio et al.^4^^,^^19^ Hypercalcemia is likely of a multifactorial etiology.

The first suggested mechanism was macrophage differentiation to mononucleated cell granulomas that secrete cytokines, which stimulate osteoclasts.^4^ The second suggestion was elevated PTH-released peptide (PTHrP), which activated renal 1-α hydroxylase and increased 1,25 dihydroxy vitamin D levels, subsequently increasing calcium absorption from the gut and bones.^20^ Elevated PTHrP levels are mostly reported in malignancy; however, it has been reported in other conditions, such as granulomatous diseases like sarcoidosis, Serratia infection, and PH.^4^^,^^21^^,^^22^ Fierer et al^23^ detected PTHrP expression in granuloma cells in both hypercalcemic and nonhypercalcemic patients, regardless of the granuloma etiology. Indeed, only Murad et al^4^ reported increased serum PTHrP levels in a 35-year-old male patient with PH1. The remainder PH cases did not evaluate PTHrP levels.^23^

Our patient showed extensive bone lesions, hypoparathyroidism, and hypercalcemia. Hypercalcemia seemed to be a late manifestation of PH, occurring when bones were extensively infiltrated by CaOx deposits, accompanied by an epithelioid and giant cell foreign body reaction, which could directly mediate in situ osteoclast activation.

To the best of our knowledge, hypothyroidism was described in 7 cases complicating PH in the literature.^4^^,^^7^ The thyroid ultrasound in these cases ranged from homogenous normal to diffuse microcalcifications throughout the gland, in which CaOx depositions were suspected, however, no biopsy was undertaken.^4^^,^^24^ Our patient had hypothyroidism with cystic and calcified nodules in the left thyroid lobe; however, when the diagnosis was made by biopsies from other sites, no further thyroid biopsy was performed. Also, combined hypothyroidism and hypoparathyroidism suggested CaOx infiltration in thyroid and parathyroid tissues, which could result in energy metabolism defects and reduce the production of these hormones.^4^

Anemia related to PH, which is resistant to erythropoietin, has been described and is mostly accompanied by debilitating bone pain, generalized skeletal osteosclerosis, osteopenia, and deformities.^4^^,^^7^^,^^12^^,^^25^^,^^26^ Indeed, anemia induced by PH1 was reported in a few case reports, and radiographs showed diffuse lytic and resorptive areas including long bones and spine accompanied by diffuse soft tissue calcifications.^4^^,^^7^^,^^25^ Anemia developed secondary to extensive bone marrow replacement by CaOx crystals infiltration, fibrosis, and foreign body giant cell reaction.^4^^,^^7^^,^^25^ Of note, elevated ferritin levels were described in a few patients.^1^^,^^4^^,^^7^^,^^25^

The current patient experienced anemia resistant to erythropoietin and iron supplements with several previous blood transfusions accompanied by elevated ferritin. The misdiagnosis of bone pain as renal osteodystrophy and the negative family history of PH could be the reason for delay the diagnosis in this patient. Thereafter, neurologic symptoms in addition to MRI findings and under open surgery observation, the diagnosis was defined by vertebral and bone marrow biopsies. In addition to this case, reviewing the literature showed only 6 patients who were diagnosed with PH by bone marrow biopsies.^1^^,^^3^^,^^7^^,^^10^^,^^18^^,^^25^

A laboratory diagnosis could be made based on measurements of urinary oxalate (Uox) excretion and plasma oxalate (Pox) levels. Uox levels are less than 0.5 mmol/1.73 m^2^/day in normal individuals and more than 1 mmol/1.73 m^2^/day in patients with PH.^6^ Patients with PH have significantly higher Pox levels (>80 mmol/L).^27^ In the presence of kidney failure without PH, Pox levels are elevated due to decreased excretion.^27^ A definitive diagnosis is possible by molecular testing for causative PH genes (AGXT, GRHPR, and HOGA1).^5^^,^^6^

The cause of our patient’s kidney failure was PH with nephrocalcinosis aligned with no residual urinary output, and the patient received 2 regular HD sessions per week. The measurement of Pox levels from the beginning of the kidney failure diagnosis and further receiving intensified HD sessions could help in altering the course of the disease and not reaching to this progressed status. However, the delay in PH diagnosis and receiving only 2 HD sessions represented the main reasons for this devastating condition. So, clinicians should always investigate the cause of kidney failure in an attempt to modify or alleviate the course of such disorders as in this case.

There is heterogeneity of disease expression in all 3 PH types. The clinical and age of presentation are variable even among family members with the same genotype.^6^ This translates with an age at diagnosis ranging from less than 1 year to over 50 years.^6^^,^^28^ The definitive diagnosis is essentially made by genetic testing; however, some previous reports (Table 2) guide the diagnosis first by biopsies from the affected organs and then confirmed this using molecular testing.^3^^,^^10^^,^^14^^,^^15^^,^^17^^,^^18^ Ardemani et al^11^ made the diagnosis of PH1 by cranial ultrasound and ophthalmoscopy, which revealed retinal crystals in both maculae without genetic testing. Other reports (Table 2) mentioned the systemic CaOx depositions in affected organs (skin, sural nerve, bone) and then testing the mutations. PH1 was reported in 5 patients (3,14,15,17,18), and PH2 in 1 patient.^10^ PH1 is the most common (70%-80%) and the most severe infiltrative form.^6^ Although genetic testing was not available in our case, this severe systemic infiltrative disease suggested the PH1 form.

The standard treatment for PH included hydration, urinary alkalinization, and pyridoxine (vitamin B6), the latter in PH1.29 When the glomerular filtration rate is less than 30 mL/min per 1.73 m^2^, most patients need kidney replacement therapy (dialysis or transplantation).^30^ However, no effective dialysis model could adequately remove the endogenous oxalate overproduction, frequent HD sessions (5-6 days weekly) with nightly peritoneal dialysis could help in minimizing the systemic CaOx precipitation, where the postdialysis Pox level of <30 μmol/L should be achieved as possible.^30^ Liver transplantation remains the only cure for patients with PH1, which is combined with kidney transplantation for PH1 patients and kidney failure.^29^ On the other hand, isolated kidney transplantation is the transplant method of choice for patients with PH2.^29^ However, the follow-up of a small group of PH2 patients, who received kidney transplantation, showed graft dysfunction induced by CaOx precipitation, which makes subsequent liver transplantation necessary.^29^^,^^30^ In addition, no transplantation data are currently available for patients with PH3.^29^^,^^30^

New therapies are available for PH, including 2 RNAi therapies, lumasiran and nedosiran.^29^^,^^30^ Both drugs showed highly effective in lowering endogenous oxalate production in patients with PH1; however, no conclusive data were seen in patients with PH2 or PH3.^29^^,^^30^ These RNAi therapies showed a trend toward better clinical outcomes, including a lower rate of kidney stone events, an improvement in nephrocalcinosis, a better preservation of kidney function, and prevention of progression to kidney failure and systemic oxalosis.^30^ However, both drugs were not successful in patients in whom systemic oxalosis already existed.^30^

In this case, although the patient came in a progressive and late stage of systemic oxalosis, only 2 stone events were reported before. The patient was not evaluated for the definitive cause of kidney failure and was not evaluated for kidney transplantation before. This resulted in this patient's devastating status. Unfortunately, there was no definitive or beneficial treatment could be applied for the patient at this progressed stage. Clinicians should always consider PH in the differential diagnosis of kidney failure patients with stone events because the early diagnosis of PH could be lifesaving.

## Conclusion

We reported rare and devastating manifestations of PH in a patient with kidney failure. CaOx infiltration caused spinal cord compression because of vertebral destruction and mass-like lesions. Additionally, multiorgan CaOx infiltration caused resistant anemia, hepatosplenomegaly, extensive bone destruction, hypoparathyroidism, hypothyroidism, and hypercalcemia. The misdiagnosis of bone pain as renal osteodystrophy and the negative family history could delay the diagnosis of PH in patients with kidney failure. Clinicians should have a low threshold to search for the PH suspicion in patients with kidney failure that is associated with kidney stone events. Bone marrow biopsy or other affected organs could help in PH diagnosis.
